# Supplementary material for: Biomechanical analysis of iliosacral and transiliac–transsacral screw combinations for fixation of undisplaced Denis II vertical shear fractures in dysmorphic sacrum
Source: PeerJ. 2025 Oct 10;13:e20139. doi: 10.7717/peerj.20139 (PMC12517282; doi:10.7717/peerj.20139)
Supplement: Supplemental Information 1 [file peerj-13-20139-s001.docx]

**Supplementary 1 Table 1**  Under forward flexion conditions, the maximum Von Mises stress and deformation of the S1 and S2 segment screws.

|  | Maximum Von Mises stress  of the screw (MPa) | |  | Deformation of the  screw (mm) | |
| --- | --- | --- | --- | --- | --- |
|  | S1 segment | S2 segment |  | S1 segment | S2 segment |
| G1 | 135.16 | - |  | 0.84 | - |
| G2 | - | 100.15 |  | - | 0.46 |
| G3 | 120.10 | 65.97 |  | 0.82 | 0.45 |
| G4 | - | 85.53 |  | - | 0.23 |
| G5 | 69.14 | 66.17 |  | 0.41 | 0.16 |
| G6 | 63.32 | 65.77 |  | 0.50 | 0.20 |
| G7 | 75.77 | 60.10 |  | 0.49 | 0.19 |

**Supplementary 1 Table 2** Under left flexion conditions, the maximum Von Mises stress and deformation of the S1 and S2 segment screws.

|  | Maximum Von Mises stress  of the screw (MPa) | |  | Deformation of the  screw (mm) | |
| --- | --- | --- | --- | --- | --- |
|  | S1 segment | S2 segment |  | S1 segment | S2 segment |
| G1 | 101.41 | - |  | 0.70 | - |
| G2 | - | 73.80 |  | - | 0.37 |
| G3 | 90.36 | 61.03 |  | 0.69 | 0.43 |
| G4 | - | 58.83 |  | - | 0.19 |
| G5 | 48.56 | 66.70 |  | 0.29 | 0.15 |
| G6 | 39.86 | 67.87 |  | 0.35 | 0.18 |
| G7 | 48.67 | 64.21 |  | 0.35 | 0.18 |

**Supplementary 1 Table 3** Under right flexion conditions, the maximum Von Mises stress and deformation of the S1 and S2 segment screws.

|  | Maximum Von Mises stress  of the screw (MPa) | |  | Deformation of the  screw (mm) | |
| --- | --- | --- | --- | --- | --- |
|  | S1 segment | S2 segment |  | S1 segment | S2 segment |
| G1 | 98.08 | - |  | 0.56 | - |
| G2 | - | 102.76 |  | - | 0.32 |
| G3 | 80.90 | 44.23 |  | 0.53 | 0.29 |
| G4 | - | 58.83 |  | - | 0.19 |
| G5 | 64.96 | 63.58 |  | 0.31 | 0.14 |
| G6 | 55.32 | 54.15 |  | 0.36 | 0.17 |
| G7 | 59.67 | 52.91 |  | 0.36 | 0.17 |

**Supplementary 1 Table 4**  Under left rotation conditions, the maximum Von Mises stress and deformation of the S1 and S2 segment screws.

|  | Maximum Von Mises stress  of the screw (MPa) | |  | Deformation of the  screw (mm) | |
| --- | --- | --- | --- | --- | --- |
|  | S1 segment | S2 segment |  | S1 segment | S2 segment |
| G1 | 57.16 | - |  | 0.36 | - |
| G2 | - | 92.18 |  | - | 0.33 |
| G3 | 87.52 | 45.42 |  | 0.55 | 0.32 |
| G4 | - | 80.45 |  | - | 0.21 |
| G5 | 57.68 | 64.12 |  | 0.31 | 0.15 |
| G6 | 49.14 | 48.87 |  | 0.37 | 0.18 |
| G7 | 57.16 | 45.45 |  | 0.36 | 0.18 |

**Supplementary 1 Table 5**  Under right rotation conditions, the maximum Von Mises stress and deformation of the S1 and S2 segment screws.

|  | Maximum Von Mises stress  of the screw (MPa) | |  | Deformation of the  screw (mm) | |
| --- | --- | --- | --- | --- | --- |
|  | S1 segment | S2 segment |  | S1 segment | S2 segment |
| G1 | 206.12 |  |  | 1.07 |  |
| G2 |  | 150.88 |  |  | 0.65 |
| G3 | 191.34 | 123.81 |  | 1.08 | 0.61 |
| G4 |  | 90.77 |  |  | 0.22 |
| G5 | 72.31 | 64.80 |  | 0.33 | 0.14 |
| G6 | 63.08 | 72.76 |  | 0.42 | 0.18 |
| G7 | 69.47 | 63.25 |  | 0.42 | 0.17 |
