# Supplementary material for: Biomechanical analysis of iliosacral and transiliac–transsacral screw combinations for fixation of undisplaced Denis II vertical shear fractures in dysmorphic sacrum
Source: PeerJ. 2025 Oct 10;13:e20139. doi: 10.7717/peerj.20139 (PMC12517282; doi:10.7717/peerj.20139)
Supplement: Supplemental Information 2 [file peerj-13-20139-s002.docx]

**Supplementary 2 Table 1** Under forward flexion conditions, the vertical displacement and sagittal plane angular displacement of the superior surface of the S1.

|  | Vertical displacement of the superior  surface of S1 (mm) | | | The superior surface of S1 in the sagittal plane angular displacement (degree) |
| --- | --- | --- | --- | --- |
|  | Min | Max | Mean |  |
| G1 | 0.5626 | 0.9347 | 0.7482 | 4.2863 |
| G2 | 0.6321 | 1.0232 | 0.8285 | 7.7410 |
| G3 | 0.5066 | 0.9069 | 0.7067 | 2.0727 |
| G4 | 0.3948 | 0.7081 | 0.5497 | 4.6025 |
| G5 | 0.2819 | 0.5110 | 0.3961 | 1.7416 |
| G6 | 0.3543 | 0.6123 | 0.4843 | 2.8408 |
| G7 | 0.3187 | 0.5614 | 0.4392 | 2.7304 |

**Supplementary 2 Table 2** Under left flexion conditions, the vertical displacement and sagittal plane angular displacement of the superior surface of the S1.

|  | Vertical displacement of the superior  surface of S1 (mm) | | | The superior surface of S1 in the sagittal plane angular displacement (degree) |
| --- | --- | --- | --- | --- |
|  | Min | Max | Mean |  |
| G1 | 0.4429 | 0.7184 | 0.6230 | 3.7874 |
| G2 | 0.4847 | 0.7910 | 0.6320 | 4.7602 |
| G3 | 0.4104 | 0.8041 | 0.6011 | 1.7578 |
| G4 | 0.2973 | 0.4714 | 0.3832 | 2.8308 |
| G5 | 0.2094 | 0.3774 | 0.2921 | 0.2788 |
| G6 | 0.2601 | 0.4466 | 0.3525 | 0.3606 |
| G7 | 0.2392 | 0.4142 | 0.3259 | 0.5798 |

**Supplementary 2 Table 3** Under right flexion conditions, the vertical displacement and sagittal plane angular displacement of the superior surface of the S1.

|  | Vertical displacement of the superior  surface of S1 (mm) | | | The superior surface of S1 in the sagittal plane angular displacement (degree) |
| --- | --- | --- | --- | --- |
|  | Min | Max | Mean |  |
| G1 | 0.4238 | 0.6173 | 0.5158 | 1.3774 |
| G2 | 0.4720 | 0.7416 | 0.6020 | 2.2331 |
| G3 | 0.3776 | 0.5608 | 0.4684 | 0.7281 |
| G4 | 0.2973 | 0.4714 | 0.3832 | 1.6891 |
| G5 | 0.2127 | 0.3797 | 0.2951 | 0.6141 |
| G6 | 0.2631 | 0.4491 | 0.3547 | 0.6821 |
| G7 | 0.2464 | 0.4275 | 0.3352 | 0.6421 |

**Supplementary 2 Table 4** Under left rotation conditions, the vertical displacement and sagittal plane angular displacement of the superior surface of the S1.

|  | Vertical displacement of the superior  surface of S1 (mm) | | | The superior surface of S1 in the sagittal plane angular displacement (degree) |
| --- | --- | --- | --- | --- |
|  | Min | Max | Mean |  |
| G1 | 0.4301 | 0.6407 | 0.5375 | 2.1783 |
| G2 | 0.4772 | 0.7107 | 0.5957 | 3.5631 |
| G3 | 0.3783 | 0.6022 | 0.4915 | 3.2098 |
| G4 | 0.3118 | 0.5263 | 0.4192 | 2.3366 |
| G5 | 0.2289 | 0.3589 | 0.2942 | 1.4012 |
| G6 | 0.2872 | 0.4387 | 0.3642 | 1.6287 |
| G7 | 0.2616 | 0.4005 | 0.3310 | 1.4675 |

**Supplementary 2 Table 5** Under right rotation conditions, the vertical displacement and sagittal plane angular displacement of the superior surface of the S1.

|  | Vertical displacement of the superior  surface of S1 (mm) | | | The superior surface of S1 in the sagittal plane angular displacement (degree) |
| --- | --- | --- | --- | --- |
|  | Min | Max | Mean |  |
| G1 | 0.6355 | 1.1279 | 0.8795 | 18.5930 |
| G2 | 0.7337 | 1.2876 | 1.0185 | 22.7270 |
| G3 | 0.5879 | 1.1308 | 0.8556 | 15.7330 |
| G4 | 0.3672 | 0.6330 | 0.5039 | 9.8009 |
| G5 | 0.2424 | 0.4070 | 0.3269 | 3.1175 |
| G6 | 0.3222 | 0.5205 | 0.4252 | 4.8906 |
| G7 | 0.2870 | 0.4665 | 0.3786 | 4.3542 |
