# Supplementary material for: Biomechanical analysis of iliosacral and transiliac–transsacral screw combinations for fixation of undisplaced Denis II vertical shear fractures in dysmorphic sacrum
Source: PeerJ. 2025 Oct 10;13:e20139. doi: 10.7717/peerj.20139 (PMC12517282; doi:10.7717/peerj.20139)
Supplement: Supplemental Information 3 [file peerj-13-20139-s003.docx]

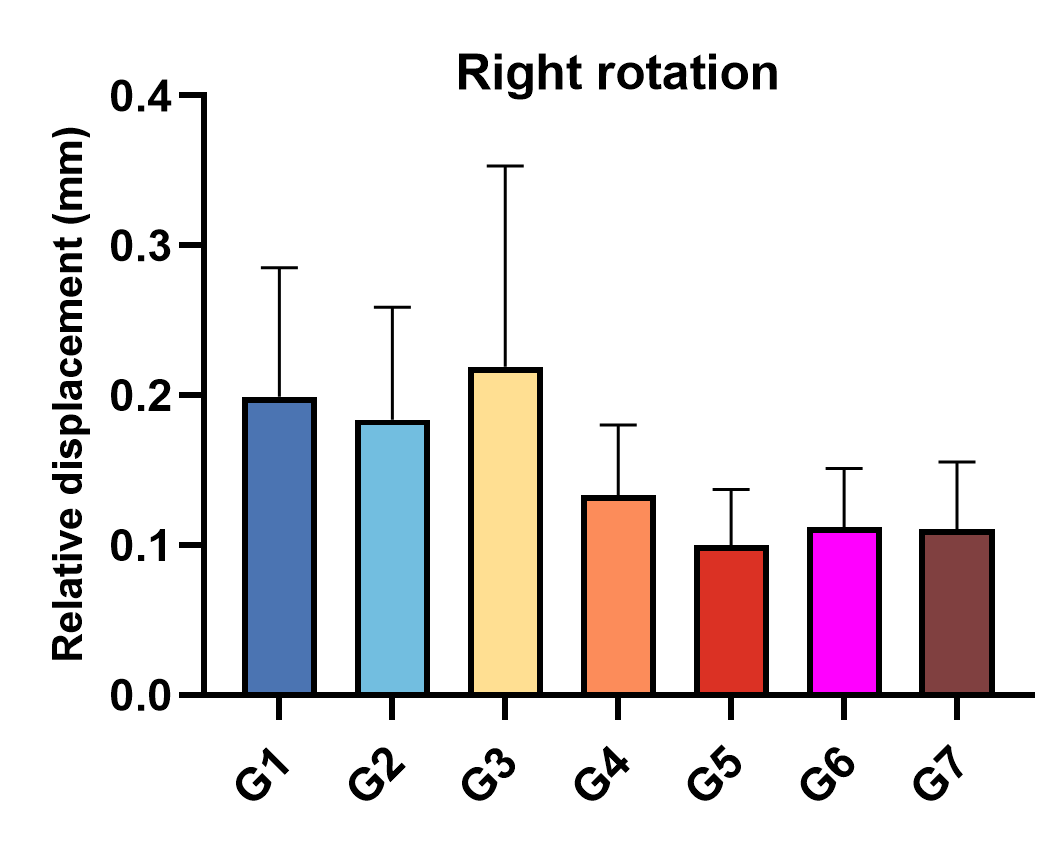

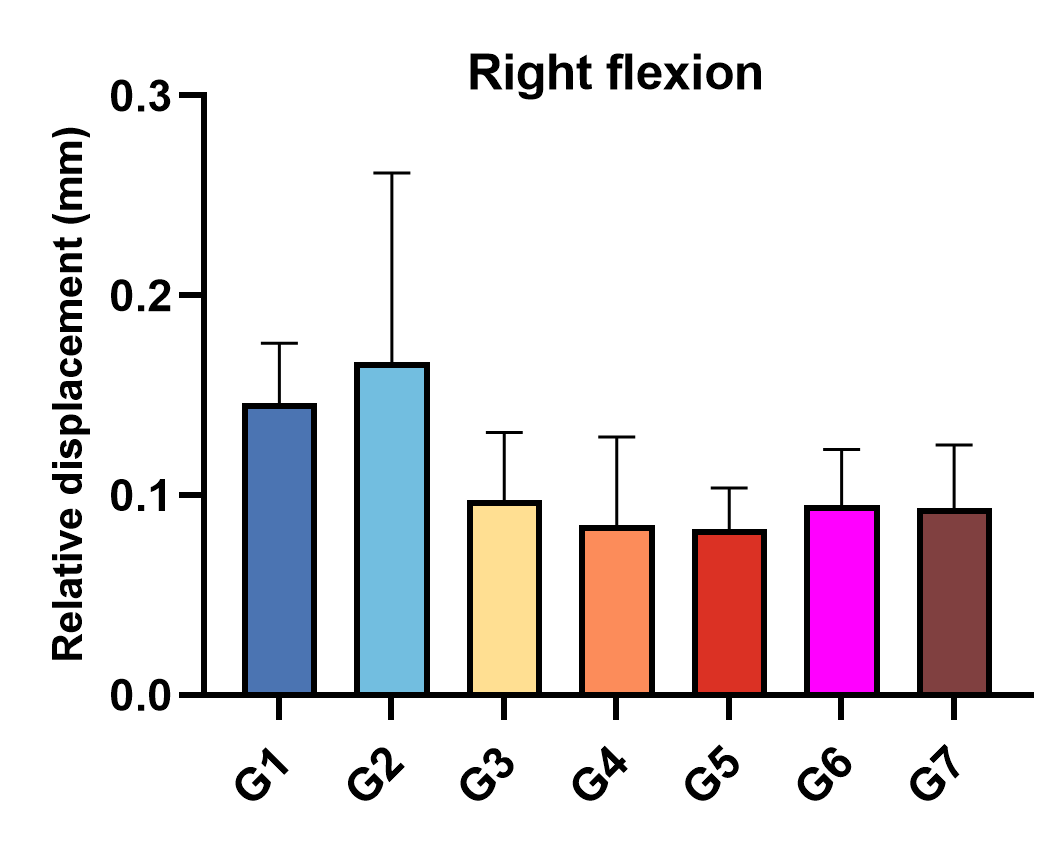

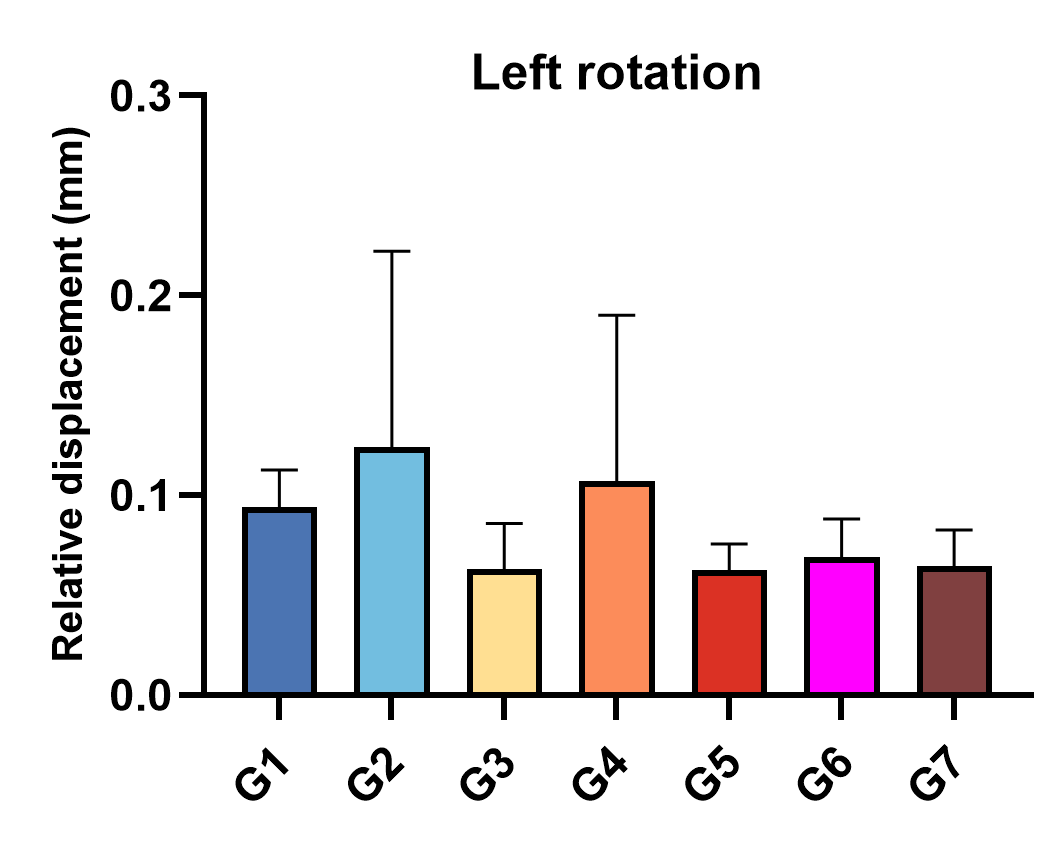

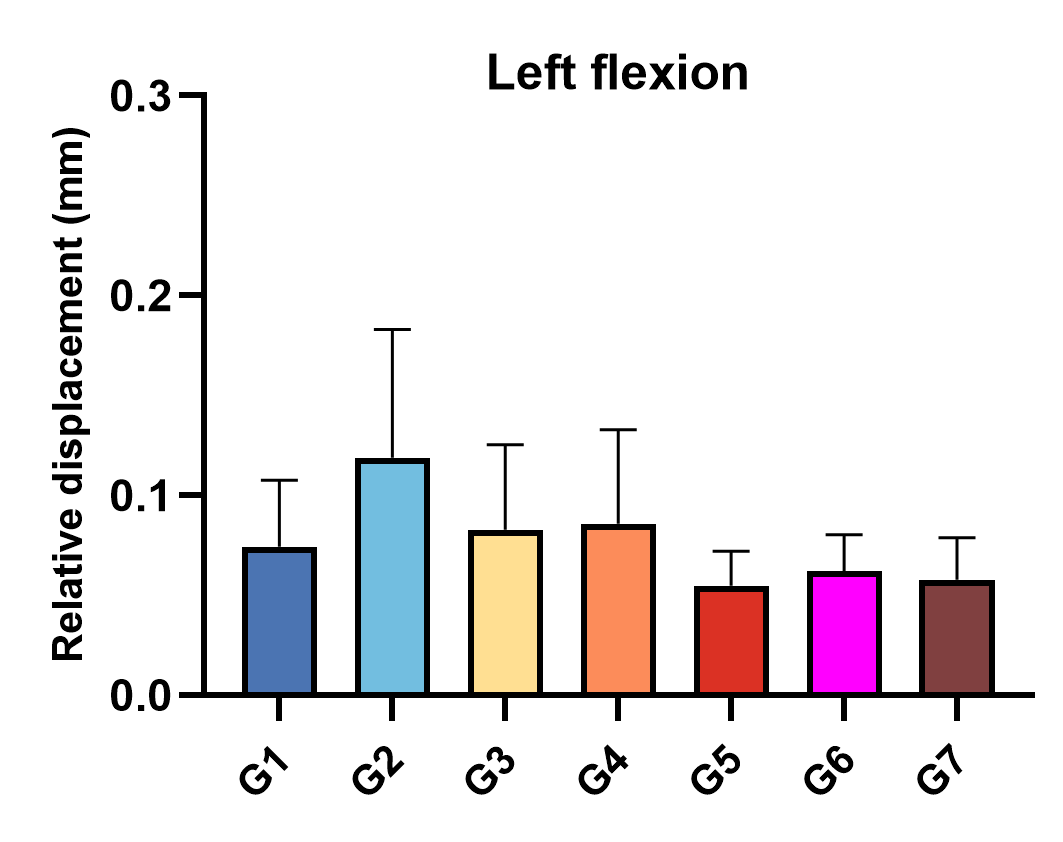

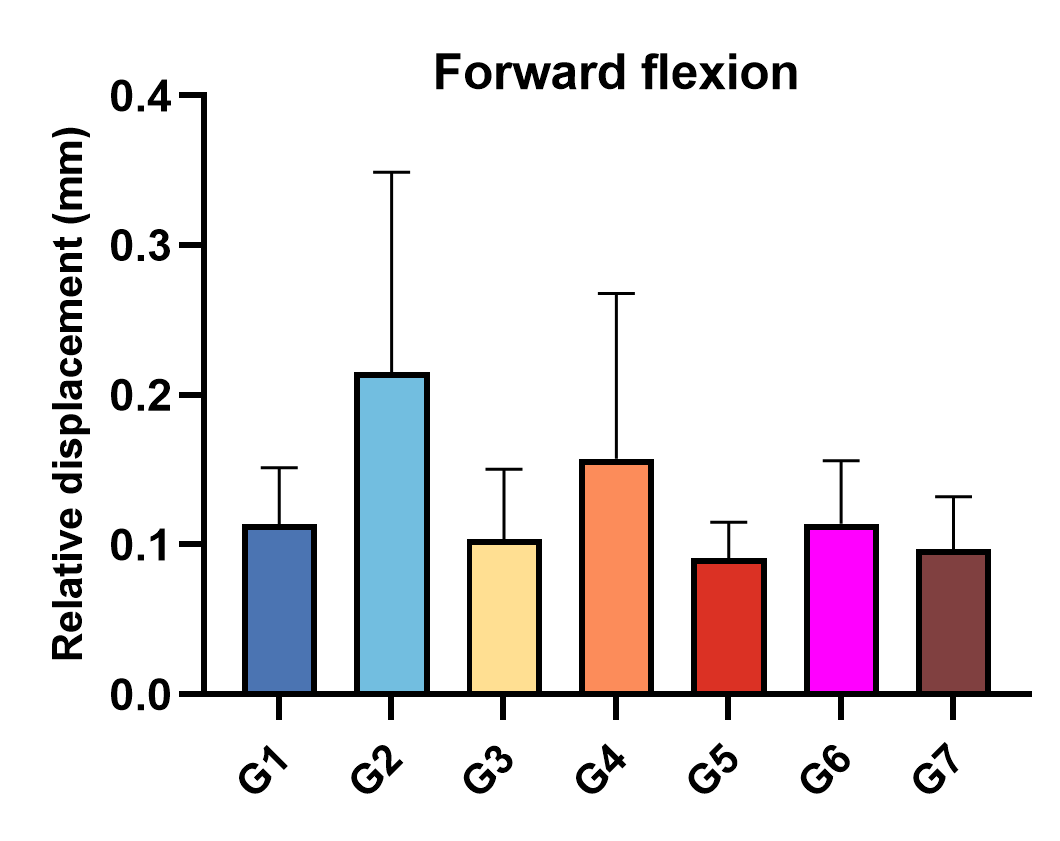
**Supplementary 3 Figure 1:** Comparison of the relative displacement on the anterior surface of the sacral under seven fixed conditions while forward flexion, left flexion, right flexion, left rotation, and right rotation.
