# Supplementary material for: Biomechanical analysis of iliosacral and transiliac–transsacral screw combinations for fixation of undisplaced Denis II vertical shear fractures in dysmorphic sacrum
Source: PeerJ. 2025 Oct 10;13:e20139. doi: 10.7717/peerj.20139 (PMC12517282; doi:10.7717/peerj.20139)
Supplement: Supplemental Information 5 [file peerj-13-20139-s005.docx]

All 44 MorphoSource DOIs associated with the 3D scan data referenced in the manuscript:

1. 10.17602/M2/M779414

2. 10.17602/M2/M778839

3. 10.17602/M2/M778570

4. 10.17602/M2/M778521

5. 10.17602/M2/M778512

6. 10.17602/M2/M778503

7. 10.17602/M2/M778502

8. 10.17602/M2/M778500

9. 10.17602/M2/M778498

10. 10.17602/M2/M778497

11. 10.17602/M2/M778447

12. 10.17602/M2/M778442

13. 10.17602/M2/M778434

14. 10.17602/M2/M778429

15. 10.17602/M2/M778424

16. 10.17602/M2/M778415

17. 10.17602/M2/M778387

18. 10.17602/M2/M778386

19. 10.17602/M2/M778381

20. 10.17602/M2/M778380

21. 10.17602/M2/M778364

22. 10.17602/M2/M778363

23. 10.17602/M2/M778362

24. 10.17602/M2/M778319

25. 10.17602/M2/M778320

26. 10.17602/M2/M778317

27. 10.17602/M2/M778316

28. 10.17602/M2/M778315

29. 10.17602/M2/M778285

30. 10.17602/M2/M778275

31. 10.17602/M2/M778262

32. 10.17602/M2/M778261

33. 10.17602/M2/M778260

34. 10.17602/M2/M778240

35. 10.17602/M2/M778239

36. 10.17602/M2/M778225

37. 10.17602/M2/M778216

38. 10.17602/M2/M778201

39. 10.17602/M2/M778191

40. 10.17602/M2/M778190

41. 10.17602/M2/M778179

42. 10.17602/M2/M778172

43. 10.17602/M2/M778162

44. 10.17602/M2/M777711
